# Synergistic chemo-immunotherapy for osteosarcoma via a pH-responsive multi-component nanoparticle system

**DOI:** 10.3389/fphar.2025.1584245

**Published:** 2025-04-08

**Authors:** Dapeng Li, Yuanfan Li, Jie Cang, Xianwen Yan, Feipeng Wu, Xuan Sun, Wenchao Zhang

**Affiliations:** ^1^ Department of Spine Surgery, Affiliated Hospital of Jiangsu University, Zhenjiang, Jiangsu, China; ^2^ School of Medicine, Jiangsu University, Zhenjiang, Jiangsu, China; ^3^ School of Pharmacy, Jiangsu University, Zhenjiang, Jiangsu, China; ^4^ Department of Orthopedics, Affiliated Jintan Hospital of Jiangsu University, Jintan, Jiangsu, China

**Keywords:** osteosarcoma, pH-responsive immune-modulating nanoparticles, tumor microenvironment reprogramming, immunogenic cell death, TLR4 and STING agonists

## Abstract

**Introduction:**

Osteosarcoma (OS) is the most common primary malignant bone tumor in pediatric populations. Its treatment is complicated by chemotherapy-induced toxicity and limited induction of immunogenic cell death (ICD).

**Methods:**

To address these challenges, we developed a pH-responsive, multi-component nanoparticle system designed to co-deliver doxorubicin (DOX), monophosphoryl lipid A (MPLA), and a PD-1/PD-L1-targeting peptide, integrated with the immune-modulating polymer PEG-PC7A. The system was optimized using both one-factor-at-a-time (OFAT) and Box-Behnken design (BBD).

**Results:**

The optimized nanoparticles had a hydrodynamic size of 110 nm, high encapsulation efficiency (97.15%), and pH-sensitive drug release (91% at pH 6.5). In vitro studies showed enhanced ICD markers, including calreticulin exposure and ATP/HMGB1 release, aswell as synergistic dendritic cell maturation via dual STING/TLR4 pathway activation. In an orthotopic LM8 osteosarcoma model, the nanoparticles significantly suppressed tumor growth, promoted cytotoxic T lymphocyte infiltration, reduced regulatory T cells, and established long-term immune memory.

**Discussion:**

The combination of ICD induction, innate immune activation, and checkpoint blockade reprogrammed the tumor microenvironment, amplifying anti-tumor immune responses. These results demonstrate the potential of this multifunctional nanoparticle platform as an effective immunochemotherapeutic strategy for osteosarcoma, offering enhanced therapeutic efficacy and reduced systemic toxicity.

## 1 Introduction

Osteosarcoma (OS) is the most common primary malignant bone tumor (Beird et al., 2022), predominantly affecting pediatric and adolescent populations, accounting for over 50% of childhood bone malignancies. Despite advancements in treatment, including a combination of chemotherapy and surgery, the long-term prognosis remains a challenge (Beird et al., 2022). Over the past few decades, the 5-year survival rate has improved from less than 20% to approximately 60%–80%. However, treatment strategies remain highly aggressive and accompanied by severe adverse effects such as toxicity, secondary malignancies, and diminished quality of life (Panez-Toro et al., 2023).

A major challenge in OS treatment lies in the toxicity associated with conventional chemotherapeutic agents. Doxorubicin is highly effective in inducing cytotoxicity in the treatment of osteosarcoma but is also linked to dose-dependent cardiotoxicity (Chen et al., 2022), affecting approximately 30% of long-term survivors receiving cumulative doses exceeding 550 mg/m^2^ (Alberts and Garcia, 1997; Treat et al., 1990). Although reducing DOX dosage can alleviate the cardiotoxicity, this reduction risks compromising the therapeutic efficacy, particularly its ability to induce immunogenic cell death (ICD) (Casares et al., 2005; Huang et al., 2023; Jiang et al., 2021). ICD is a specialized form of apoptosis that elicits a potent anti-tumor immune response via the release of damage-associated molecular patterns (DAMPs), a process critically dependent on maintaining an optimal therapeutic dose (Galluzzi et al., 2023; Birmpilis et al., 2022; Kroemer et al., 2022). However, while ICD can enhance immune-mediated tumor clearance, many chemotherapeutic agents fail to fully induce this process, requiring additional strategies to enhance its immunogenicity.

Since chemotherapy alone is often insufficient to trigger a robust ICD response, the combination therapy integrating immune stimulatory agents has gained interest. Monophosphoryl lipid A (MPLA) (Li R. et al., 2023; Mata-Haro et al., 2007; Cui et al., 2014), a detoxified derivative of lipopolysaccharide (LPS), acts as a Toll-like receptor 4 (TLR4) agonist and has been shown to enhance dendritic cell (DC) maturation, promote the secretion of pro-inflammatory cytokines, and potentiate the activation of tumor-specific cytotoxic T cells. By integrating MPLA into chemotherapy regimens, it may be possible to amplify the immune response triggered by ICD, thereby improving overall anti-tumor efficacy. Furthermore, osteosarcoma cells frequently exploit immune checkpoint pathways to evade immune surveillance, making PD-1/PD-L1 blockade an essential strategy to enhance anti-tumor T cell responses (Lussier et al., 2015a; Lussier et al., 2015b; Chen et al., 2020). However, the therapeutic efficacy of immune checkpoint blockade (ICB) in OS remains inconsistent due to the heterogeneous and dynamic expression of PD-L1 within the tumor microenvironment (Panez-Toro et al., 2023; Tawbi et al., 2017; Boye et al., 2021; Le Cesne et al., 2019). For instance, SARC028 trial (NCT02301039) was performed to assess the activity of anti-PD-1 antibodies in the treatment of soft-tissue sarcoma and bone sarcoma (Tawbi et al., 2017), which resulted in only 5% of patients with bone sarcoma having an objective response. These low response rates were due to the immune-cold nature of OS tumors and PD-L1 heterogeneity. To overcome this limitation, PD-1/PD-L1-targeting peptides can be utilized alongside ICD-inducing agents to synergistically enhance immune activation and inhibit tumor immune evasion mechanisms (Rui et al., 2023). Therefore, a strategy containing chemotherapeutic agent, TLR-4 agonist, and PD-1/PD-L1-targeting peptide would effectively enhance the anti-tumor immunity and inhibit the aggressive growth of OS. However, the effective co-delivery of these components remains a significant challenge, showing the need for an optimized drug delivery system to ensure precise co-administration and synergistic therapeutic effects.

**SCHEME 1:**
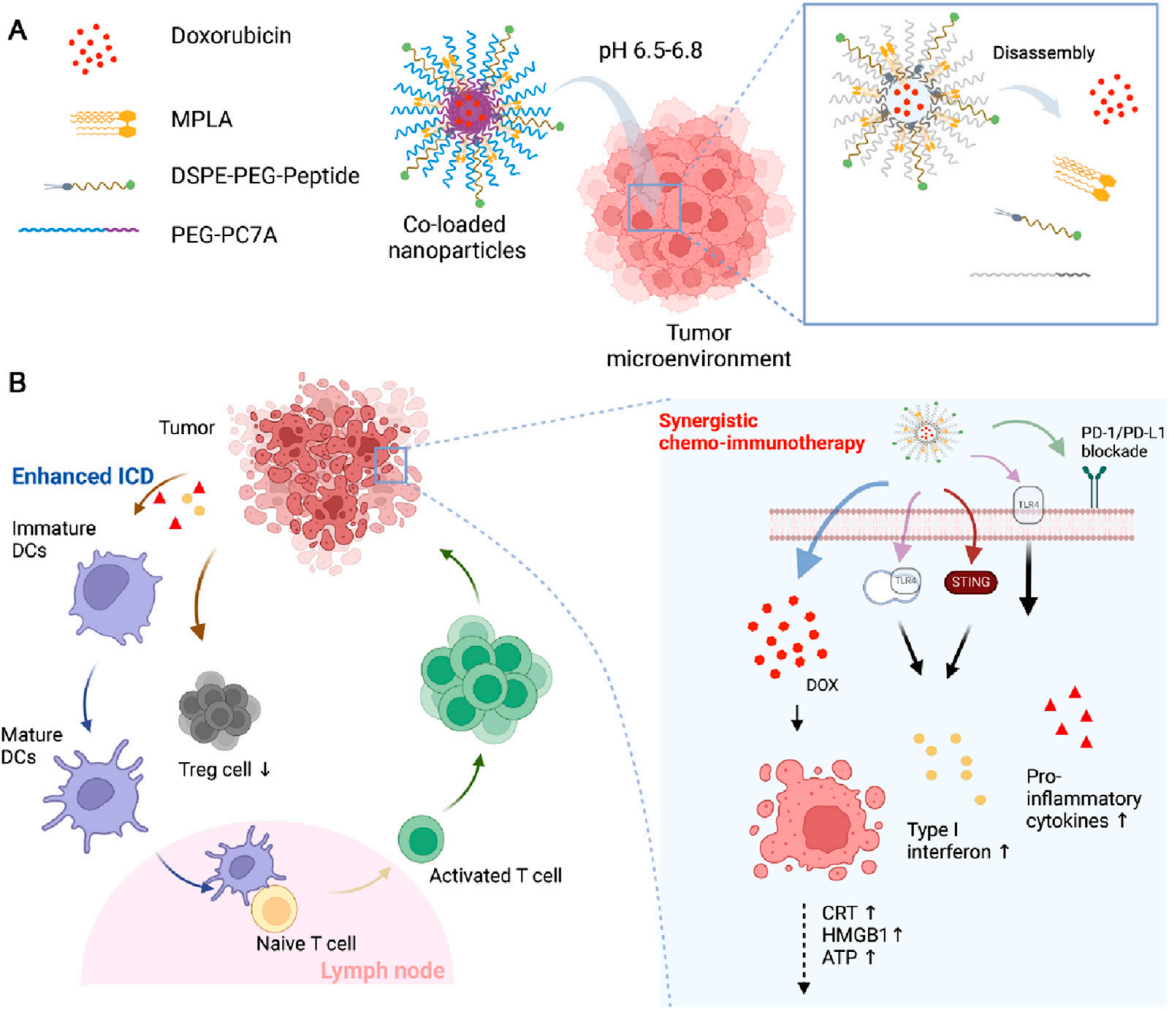
Scheme illustration of the multi-component co-loaded nanoparticles, as well as the mechanism of chemo-immunotherapy. **(A)** The structure of co-loaded nanoparticles. **(B)** The synergistic chemo-immunotherapy of the co-loaded nanoparticles.

To effectively maximize the synergistic therapeutic effects of this combination strategy, we have developed a multi-component nanoparticle system encapsulating doxorubicin, MPLA, and a PD-1/PD-L1-targeting peptide (Scheme 1). This delivery system employs PEG-PC7A, a pH-sensitive polymer with well-established immune-modulating properties (Yang et al., 2023; Cheng et al., 2021), to enable controlled drug release and enhance immune activation. PEG-PC7A facilitates pH-responsive drug release, ensuring site-specific activation within the tumor microenvironment while regulating immune responses through the cyclic GMP-AMP synthase (cGAS)-stimulator of interferon genes (STING) pathway (Li et al., 2024; Wang et al., 2021; Li Y. et al., 2023). By integrating chemotherapy-induced ICD, innate immune activation via TLR4 stimulation, and adaptive immune enhancement through checkpoint blockade, this nanoparticle-based strategy could provide a comprehensive and synergistic approach to improving osteosarcoma treatment outcomes.

In this study, the therapeutic potential of this multi-functional nanoparticle system was comprehensively evaluated in in vitro cellular assays and *in vivo* osteosarcoma model. We investigate its ability to induce ICD-mediated immune responses, enhance dendritic cell activation and antigen presentation, promote T cell infiltration and tumor regression.

## 2 Materials and methods

### 2.1 Materials

Doxorubicin hydrochloride (DOX) was obtained from Sinopharm Chemical Reagent Co., Ltd. (Shanghai, China). Monophosphoryl lipid A (MPLA) was purchased from Sigma-Aldrich Corp. (St. Louis, MO). MeO-PEG_114_-Br, N,N,N′,N″,N″-pentamethyldiethylenetriamine (PMDETA), 2-propanol, dimethylformamide (DMF), CuBr, tetrahydrofuran (THF), Al_2_O_3_, triethanolamine hydrochloride (TEA•HCl), sodium phosphate, NaCl, and ethylenediaminetetraacetic acid (EDTA) were purchased from Aladdin Reagent Co. (Shanghai, China). 1,2-distearoyl-sn-glycero-3-phosphoethanolamine-N-[carbonyl-amino(polyethylene glycol)-2000-N’-(3-maleimidopropionyl)] (ammonium salt) (DSPE-PEG-MAL) was obtained from Xi’an Ruixi Biological Technology Co., Ltd (Shanxi, China). The PD-1/PD-L1 targeting peptide (sequence: CGGGGSHFSASYDKYAEKF, lyophilized, >90% purity) were synthesized by Genscript Inc. (Nanjing, China). The CCK-8 reagent was purchased from Beyotime Biotechnology (C0042, Jiangsu, China).

### 2.2 Synthesis of PEG-PC7A

The pH-sensitive polymer PEG-PC7A was synthesized via an atom transfer radical polymerization method following the previous paper (Li et al., 2021). Initially, monomer 2-hexamethyleneiminoethyl methacrylate (C7A-MA) was synthesized and purified according to the established methods (Zhou et al., 2012). To prepare PEG-PC7A, C7A-MA (3.4 g, 16 mmol), MeO-PEG_114_-Br (0.5 g, 0.1 mmol) and PMDETA (21 μL, 0.1 mmol) were dissolved in a 1:1 mixture of 2-propanol and DMF in a Schlenk flask. The solution was deoxygenated through three freeze-pump-thaw cycles before adding CuBr (14 mg, 0.1 mmol) under nitrogen protection. The polymerization reaction proceeded under vacuum at 40°C overnight. After polymerization, the reaction mixture was diluted with THF (10 mL) and purified by passage through a neutral Al_2_O_3_ column to remove residual catalyst. The solvent was evaporated, and the crude product was dialyzed against distilled water before lyophilization, yielding a white powder. The purified polymer PEG-PC7A was characterized using ^1^H NMR spectroscopy to confirm its structure. The ^1^H NMR spectrum of resultant polymer was presented as Supplementary Figure S1.

### 2.3 Conjugation of PD-1/PD-L1 targeting peptide

A PD-1/PD-L1 targeting peptide (CGGGGSHFSASYDKYAEKF), previously reported to block the PD-1/PD-L1 immune checkpoint (Rui et al., 2023), was conjugated to lipid DSPE-PEG-Mal to facilitate the formulation of co-loaded nanoparticles. Briefly, DSPE-PEG-Mal (2 mM) and the peptide (4 mM) were dissolved in a buffer solution (50 mM TEA, 50 mM sodium phosphate, 150 mM NaCl, and 1 mM EDTA, pH 8). The mixture was gently stirred overnight in a sealed glass bottle at 4°C. The synthesized DSPE-PEG-peptide product was characterized by matrix-assisted laser desorption/ionization time-of-flight (MALDI-TOF) mass spectrometry, and the resultant mass spectrum of peptide was presented as Supplementary Figure S2.

### 2.4 Formulation design and optimization of multi-component co-loaded nanoparticles

#### 2.4.1 Preparation and characterization of co-loaded nanoparticles

The co-loaded nanoparticles were designed to synergistically deliver DOX, MPLA, PEG-PC7A, and DSPE-PEG-peptide. Briefly, doxorubicin hydrochloride (DOX•HCl) was converted into its free base form (DOX) by stirring overnight with a two-fold molar excess of triethylamine in DMSO. Subsequently, DOX, PEG-PC7A and MPLA were dissolved in a chloroform/methanol (3:1, v/v) mixture and subjected to rotary evaporation to remove the organic solvent and form a lipid thin film. DSPE-PEG-peptide was separately dissolved in phosphate-buffered saline (PBS, 20 mM, pH 7.4). The film obtained was then hydrated using the DSPE-PEG-peptide PBS solution, and subjected to sonication to produce nano-scaled co-loaded nanoparticles. To remove free DOX and other lipids, the nanoparticle solution was dialyzed against PBS at pH 7.4 in the dark. Finally, the purified co-loaded nanoparticles were stored at 4°C. For the preparation of blank nanoparticles, MPLA and PEG-PC7A were replaced with PEG-PLGA at the same concentration, followed by a similar preparation procedure.

The average hydrodynamic diameters sizes, polydispersity index (PDI) and zeta-potential of the co-loaded nanoparticles were determined by dynamic light scattering (DLS) using a Zetasizer Nano ZS90 (Malvern Instruments, Malvern, United Kingdom). The measurements were performed at predefined temperature and pH and repeated three times for each sample. The morphology of co-loaded nanoparticles was examined by transmission electron microscopy. Briefly, a drop of the corresponding suspension was added to on a carbon-coated copper grid for 30 s and then the samples were stained with 2% phosphotungstic acid (5 μL) for 30 s. The morphology of co-loaded nanoparticles was observed using JEM-2100 transmission electron microscope (TEM, JEOL, Japan).

After the removal of free DOX via dialysis, the DOX concentration of co-loaded nanoparticles was measured by a NanoDrop 2000 spectrophotometer (Thermo Fisher Scientific, Waltham, MA, USA) at the detection wavelength of 485 nm. The encapsulation efficiency (EE) of DOX was calculated according to the following equation:

$$EE\;\left(\%\right)=\frac{Weight\;of\;encapsulated\;DOX}{Weight\;of\;DOX\;initially\;added}\times 100\%$$

The drug loading (DL) of DOX was calculated according to the following equation:

$$DL\;\left(\%\right)=\frac{Weight\;of\;encapsulated\;DOX}{Total\;weight\;of\;nanoparticles}\times 100\%$$

#### 2.4.2 One-factor-at-a-time (OFAT) optimization studies

To optimize the nanoparticle formulation, OFAT experiments were conducted by varying the concentrations of DSPE-PEG-peptide, PEG-PC7A, MPLA, and DOX while keeping other parameters constant. The effect of DSPE-PEG-peptide concentration (0.5–3 mg) was evaluated on particle size, PDI, and encapsulation efficiency of DOX (EE). Similarly, PEG-PC7A concentrations (3–8 mg), MPLA concentrations (0.1–0.5 mg), and DOX concentration (1–4 mg) were examined to assess their influence on the same formulation responses.

#### 2.4.3 Optimization of formulation using the Box-Behnken design method

Based on OFAT method, four critical formulation variables were identified that significantly influenced nanoparticle encapsulation efficiency: DSPE-PEG-Peptide (X1), PEG-PC7A (X2), MPLA (X3), and DOX (X4). A response surface methodology (RSM) based Box-Behnken design (BBD) experimental method was subsequently employed to optimize these factors, using encapsulation efficiency (Y) as the response variable. For this purpose, the effects of four variables at three levels (low, medium, and high, denoted by coded values −1, 0, and +1, respectively) were designed, resulting in a total of 30 experimental runs.

The experimental data were subsequently analyzed using the RSM implemented in R with the rsm package. The optimized formulation was determined by fitting a quadratic regression model to identify the ideal concentrations of each component for maximizing encapsulation efficiency while maintaining stability and drug loading capacity. This approach allowed for the systematic refinement of the nanoparticle formulation to achieve optimal therapeutic performance.

### 2.5 Characterization

#### 2.5.1 Stability

To evaluate the stability of the multi-component nanoparticles, samples were incubated in two different media: PBS and PBS containing 50% fetal bovine serum (FBS). Stability was assessed at both 25°C and 37°C to simulate storage and physiological conditions. At predetermined time points, the particle size of the nanoparticles was measured to monitor changes in size distribution.

In addition to nanoparticle size stability, the retention of the encapsulated PD-1/PD-L1 peptide was analyzed over a 48-h incubation period at 37°C. Peptide content was quantified using high-performance liquid chromatography (HPLC). This analysis aimed to determine peptide degradation in PBS and FBS-containing media, considering the potential influence of protease activity present in serum.

#### 2.5.2 *In vitro* drug release study

The *in vitro* drug release profile of DOX-loaded nanoparticles was evaluated in PBS solutions at different pH levels (pH 7.4, pH 6.8, and pH 6.5) to simulate physiological and tumor microenvironment conditions. Briefly, 2 mL of the nanoparticle suspension was placed in a dialysis bag (MWCO: 10 kDa) and immersed in 50 mL of the respective release medium. The system was maintained at 37°C under gentle shaking (100 rpm) to mimic *in vivo* conditions. At predetermined time points (0, 2, 6, 12, 24, and 48 h), 1 mL of the release medium was collected and replaced with an equal volume of fresh buffer to maintain sink conditions. The amount of released doxorubicin was quantified using fluorescence spectroscopy (Ex/Em: 470/590 nm) against a standard calibration curve.

### 2.6 *In vitro* cellular assay

#### 2.6.1 Cell culture

The LM8 murine osteosarcoma cell line, derived from C3H/He mice, was purchased from HyCyte™ (Suzhou, China). LM8 cells were maintained in Dulbecco’s modified Eagle’s medium (DMEM, Gibco, NY, United States) supplemented with 10% fetal bovine serum (FBS, Gibco). Cells were cultured at 37°C in an incubator under 5% CO_2_.

#### 2.6.2 *In vitro* cell viability

LM-8 cells were seeded at a density of 1 × 10^6^ cells/mL in a 96-well plate. After 24 h incubation, the culture medium was replaced with 100 μL of medium containing serial dilutions of different treatment samples. After 48 h incubation, 10 μL of the CCK-8 reagent (Dojindo, Kyushu, Japan) was added into each well and incubated for further 2 h at 37°C. The absorbance of each sample was measured at 450 nm using a multifunction microplate reader (Biotek, VT, United States).

#### 2.6.3 *In vitro* immunogenic cell death

The immunogenic cell death (ICD) biomarkers, including calreticulin (CRT), high-mobility group box 1 (HMGB1), and adenosine triphosphate (ATP), were analyzed. LM-8 cells were seeded in 24-well plates at a density of 1 × 10^5^ cells per well, and the cells were allowed to grow to an appropriate confluence. And the cells were treated with PBS, blank nanoparticles (equivalent to 10 μM of DOX), free DOX (10 μM), MPLA + PEG-PC7A nanoparticle (equivalent to 10 μM of DOX), and multi-component co-loaded nanoparticles (10 μM of DOX) for 24 h.

After treatment, the cells were digested, collected, and washed twice with cold PBS. To determine the surface expression of CRT, the cells were blocked with CD16/32 antibody for 10 min, incubated with Alexa 488-anti-CRT antibody (EPR3924, 1:500 v/v, Abcam) for 15 min at 4°C, washed once with PBS, resuspended with PBS, and detected by flow cytometry.

On the other hand, additional sets of cells received identical treatments were employed to determine the released HMGB1 and ATP. Briefly, culture supernatant was collected and centrifuged to remove cell debris. Subsequently, the extracellular HMGB1 was quantified using an enzyme-linked immunosorbent assay (ELISA) kits (R&D systems, China), whereas the supernatant ATP was quantified using a bioluminescence kit (No. S0026, Beyotime, China) and measured by a multifunction microplate reader.

#### 2.6.4 BMDC maturation induced by co-loaded nanoparticles

Mouse bone marrow cells were isolated from the femurs and tibias of 8–12 week old C3H/HeJ mice (GemPharmatech, Nanjing, China). Red blood cells (RBCs) were lysed using RBC lysis buffer (eBioscience, United States), and the remaining bone marrow cells were cultured in complete RPMI-1640 medium supplemented with granulocyte-macrophage colony-stimulating factor (GM-CSF, 20 ng/mL, Invitrogen, MA, United States), IL-4 (20 ng/mL, Invitrogen, MA, United States), and 2-mercaptoethanol (1:1000 v/v). The culture medium was replaced every 3 days to maintain optimal growth conditions. On day 8, immature BMDCs were harvested and seeded at a density of 1 × 10^6^ cells/well in 6-well plates.

To investigate the effects of different samples on BMDC maturation, BMDCs were co-cultured with LM8 cells (1 × 10^5^ cells/well) that had been pre-treated with different samples using the Transwell system (pore size: 0.4 μm, Corning, NT, United States). Following co-culture for either 16 or 48 h, BMDCs were collected, washed twice with cold PBS, and incubated with fluorescently labeled antibody at 4°C for 30 min. Subsequently, the cells were washed three times with PBS buffer (pH 7.4), and the fluorescence intensity was detected by a Beckman Coulter Gallios™ flow cytometer. The following anti-mouse monoclonal antibodies were used from BioLegend (San Diego, CA,USA): PE-anti-MHC II (clone AF6-120.1), FITC-anti-CD11c (clone N418), PE-anti-CD80 (clone 16-10A1), and FITC-anti-CD86 (clone GL1). Flow cytometric analysis was performed by gating on MHC II/CD11c-positive populations to quantify CD80 and CD86 expression levels. Data were analyzed using FlowJo software (FlowJo, BD Biosciences).

To further assess the immunostimulatory effects of co-loaded nanoparticles, cytokine levels in the BMDC culture supernatant after BMDCs were co-cultured with LM8 cells for 16 or 48 h, the supernatant of co-cultured cells was collected. IFN-β and TNF-α levels were analyzed using ELISA kits specific to IFN-β (EK2236) and TNF-α (EK282) (Lianke, Shanghai, China) according to the manufacturer’s instructions. Absorbance values were read at 450 nm using a multifunction microplate reader.

### 2.7 *In vivo* study

#### 2.7.1 Animal model

To investigate the *in vivo* antitumor effects, male C3H/HeJ mice (8–10 weeks old) were purchased from GemPharmatech (Nanjing, China). All animal experiments were conducted with the relevant institutional guidelines of Jiangsu University, and were approved by the Institutional Animal Care And Use Committees of Jiangsu University (UJS-IACUC-2024080202).

An orthotopic LM8-bearing OS model was established by injected subcutaneously 1 × 10^7^ LM8 cells into the backs of mice. Tumor growth was allowed to proceed for approximately 7 days prior to treatment until the tumors reached a measurable size. Tumor size was monitored using a caliper, with tumor volume calculated according to the formula: (length × width^2^)/2.

The mice were randomly assigned to one of 5 experimental groups (n = 5 per group): saline control, blank nanoparticles, free DOX, MPLA + PEG-PC7A nanoparticles, and multi-component co-loaded nanoparticles. Each group received the corresponding treatment via tail vein injection, with a DOX equivalent dose of 10 mg/kg administered every other day for a total of 7 doses. Tumor volume and mouse body weight were recorded every other day during the treatment. On day 14, the mice were euthanized under anesthesia, and the tumors were excised and weighed for further analysis. The tumor was further stained with hematoxylin and eosin (H&E) after mice sacrificed.

#### 2.7.2 Flow cytometric analysis

Flow cytometry was applied to characterize the change in immune cell populations in the tumor, and tumor-draining lymph nodes (TDLNs). Excised tumors were harvested, homogenized and consecutively strained through 70-μm filters to obtain single cell suspensions, while the lymph nodes were manually dissociated through a 40-μm filter to obtain a single cell suspension. Cells were stained with fluorescent antibodies to identify immune cell pollutions.

Single-cell suspensions of TDLNs were stained with CD11c (clone N418; Biolegend), CD80 (clone 16-10A1; Biolegend), CD86 (clone GL1; Biolegend) for the analysis of mature DCs.

Single-cell suspensions of tumor tissues were stained with CD3 (clone 145-2C11; BD), CD4 (clone RM4-5; BD), CD8 (clone 53–6.7; Biolegend), CD62L (clone MEL-14; Biolegend), or CD44 (clone 1M7; Biolegend) antibodies for flow cytometry analysis. For intracellular cytokine and marker staining, after extracellular staining, cells were fixed and permeabilized with BD Cytofix/Cytoperm kit (BD Biosciences) and incubated with fluorescently conjugated antibodies against IFNγ (clone XMG1.2; Biolegend) or granzyme B (clone GB11; Biolegend) antibodies. The suspensions of tumor tissues were stained with CD3, CD4, and Foxp3 (clone MF-14; Biolegend) antibodies for the analysis of regulatory T cells. Cells were fixed with 1% PFA, resuspended in buffer and stored at 4°C until analysis. Samples were run on a Beckman Coulter Gallios™ flow cytometer and analyzed using FlowJo software.

#### 2.7.3 Western blot analysis

Western blot was performed to elucidate the PD-1/PD-L1 immune checkpoint blockade including granzyme B and IFN-γ. After centrifugation cells were dissolved in cell lysis buffer (50 mm Tris-HCl, pH 7.4, 1% Triton X-100, 0.2% sodium deoxycholate, 0.2% SDS, 1 mM sodium EDTA) supplemented with protease inhibitors (5 μg/mL leupeptin, 5 μg/mL aprotinin, and 1 mM phenylmethylsulfonyl fluoride) and protein concentrations were determined a NanoDrop 2000 spectrophotometer (Thermo Fisher Scientific, Waltham, MA, United States). Then The lysates were analyzed by SDS-PAGE followed by Western blotting with primary antibodies as follows: anti-mouse granzyme B (clone 12F9B65; Biolegend), anti-mouse IFNγ (clone H22; Biolegend), and anti-mouse GAPDH (clone 6C5; Abcam). Proper secondary HRP conjugated anti-rabbit or anti-goat (Santa Cruz Biotechnology) antibodies were used as secondary reagents.

### 2.8 Statistical analysis

Statistical analysis was performed using GraphPad Prism software, version 8. Significant differences among multiple groups were statistically evaluated using one-way analysis of variance (ANOVA) and subsequent comparisons were made with Tukey-Kramer test, where *P*-values less than 0.05 were statistically significant. Data were presented as mean ± standard deviation (SD).

## 3 Results and discussion

### 3.1 Formulation design and optimization of multi-component co-loaded nanoparticles

To enhance the efficacy of osteosarcoma treatment, we developed and optimized a multi-component co-loaded nanoparticle system that integrated chemotherapy, immune activation, and checkpoint blockade into a single delivery platform. This formulation consisted of four key components: 1) doxorubicin (DOX), an anticancer agent capable of inducing ICD; 2) monophosphoryl lipid A (MPLA), an immune adjuvant designed to activate innate immune responses; 3) DSPE-PEG-peptide, which facilitated PD-1/PD-L1 immune checkpoint blockade for enhanced antitumor immunity, with its mass spectrum shown in Supplementary Figures S2, S4) PEG-PC7A, a pH-sensitive polymer with dual functions, whose ^1^H NMR spectrum shown in Supplementary Figure S1. First, PEG-PC7A regulates nanoparticle stability and drug release, ensuring efficient co-delivery of DOX, peptide, and MPLA within the tumor microenvironment. Second, PEG-PC7A functions as a STING pathway agonist, which synergized with MPLA to increase the secretion of type I interferon, thereby amplifying immune activation and anti-tumor responses.

#### 3.1.1 Results of one-factor-at-a-time (OFAT) experimental design

In order to optimize the formulation of multi-component co-loaded nanoparticles, a series of OFAT experiments were conducted to assess the influence of various formulation components and their concentrations on key physicochemical properties, including particle size, polydispersity index (PDI), and encapsulation efficiency of DOX (EE). The factors investigated included DSPE-PEG-peptide concentration, PEG-PC7A concentration, MPLA dosage, and DOX concentration.

The concentration of DSPE-PEG-peptide varied from 0.5 mg to 3 mg to examine its effect on nanoparticle characteristics. As shown in Table 1 results indicated that as the DSPE-PEG-peptide concentration increased from 0.5 mg to 3 mg, the particle size decreased slightly while the PDI improved. The encapsulation efficacy of DOX showed a gradual increase with 2 mg being the optimal concentration for achieving good balance in particle size and encapsulation efficiency, with an EE of 84.1% ± 1.6%.

**TABLE 1:** Effects of DSPE-PEG-peptide concentration on the formulation responses.

DSPE-PEG-peptide (mg)	Size (nm)	PDI	EE (%)
0.5	128.6 ± 2.5	0.321 ± 0.125	63.5 ± 0.8
1	112.1 ± 4.3	0.282 ± 0.053	71.3 ± 2.5
2	102.8 ± 2.7	0.224 ± 0.021	84.1 ± 1.6
3	96.4 ± 5.8	0.251 ± 0.043	82.7 ± 0.4

The PEG-PC7A concentration was adjusted from 3 mg to 8 mg to determine its effect on particle characteristics. As shown in Table 2, it was found that increasing the PEG-PC7A content led to a decrease in particle size with a slight increase in PDI. The optimal concentration was determined to be 5 mg, which resulted in stable nanoparticle characteristics with minimal increase in PDI, and effective encapsulation of DOX.

**TABLE 2:** Effects of PEG-PC7A concentration on the formulation responses.

PEG-PC7A (mg)	Size (nm)	PDI	EE (%)
3	122.1 ± 5.5	0.214 ± 0.023	81.5 ± 0.5
5	104.6 ± 3.7	0.226 ± 0.017	85.3 ± 0.8
6	95.4 ± 4.3	0.252 ± 0.046	83.6 ± 0.6
8	90.7 ± 3.2	0.284 ± 0.019	83.5 ± 0.3

The MPLA concentration was tested in the range of 0.1 mg–0.5 mg. The study found that MPLA at 0.3 mg enhanced DOX EE while maintaining stable particle size and PDI. Increasing MPLA content beyond this threshold led to an increase in PDI, potentially affecting formulation stability. Therefore, 0.3 mg was identified as the optimal dosage (Table 3).

**TABLE 3:** Effects of MPLA concentration on the formulation responses.

MPLA (mg)	Size (nm)	PDI	EE (%)
0.1	105.7 ± 3.6	0.212 ± 0.014	78.2 ± 0.4
0.2	100.4 ± 5.1	0.224 ± 0.032	80.5 ± 0.3
0.3	101.7 ± 3.6	0.246 ± 0.019	81.6 ± 0.4
0.5	95.7 ± 3.2	0.383 ± 0.025	75.8 ± 0.5

The input dose of DOX was tested from 1 mg to 4 mg to determine its impact on nanoparticle properties (Table 4). With increasing DOX dosage, a slight decrease in its encapsulation efficiency was observed. The optimal DOX concentration for achieving a good balance between size and encapsulation efficiency was found to be 2 mg, resulting in an EE of 89.8% ± 0.8% and a DL of 19.3% ± 0.6%.

**TABLE 4:** Effects of DOX concentration on the formulation responses.

DOX concentration (mg)	Size (nm)	PDI	EE (%)	DL (%)
1	95.2 ± 1.3	0.195 ± 0.045	90.3 ± 1.5	10.9 ± 0.4
2	100.6 ± 3.9	0.224 ± 0.052	89.8 ± 0.8	19.3 ± 0.6
3	114.7 ± 2.9	0.258 ± 0.031	75.2 ± 0.5	21.9 ± 0.3
4	122.3 ± 3.6	0.285 ± 0.061	64.5 ± 2.3	22.8 ± 0.5

Taken together, these OFAT experiments provided valuable insight into the optimal formulation conditions for the co-loaded nanoparticles, guiding the subsequent RSM based BBD optimization.

#### 3.1.2 Statistical analysis of the BBD

Building upon the results from the OFAT experiments, four key factors that significantly influence the formulation responses of the co-loaded nanoparticles were selected: DSPE-PEG-peptide (X1), PEG-PC7A (X2), MPLA (X3), and DOX concentration (X4) (Table 5). The encapsulation efficiency (Y) was used as the response variable. A four-factor, three-level Box-Behnken design (BBD) method was employed to optimize the formulation variables and the response surface methodology required 30 experimental runs. The experimental data were summarized in Supplementary Table S1. ANOVA testing was conducted to identify the significant terms of the chose model on the responses.

**TABLE 5:** The BBD matrix.

Factors	Low level (−1)	Central level (0)	High level (+1)
X_1_ (DSPE-PEG-Peptide: mg)	1	2	3
X_2_ (PEG-PC7A: mg)	4	5	6
X_3_ (MPLA: mg)	0.2	0.3	0.4
X_4_ (DOX: mg)	1.5	2	2.5
Y (EE%)			

The second-order polynomial regression model was applied to determine the relationship between the independent variables and the response variable:

$$Y=-66.25+31.74X_{1}+20.51X_{2}+29.15X_{3}+49.40{\text{X}}_{4}-6.03X_{1}^{2}-1.94X_{2}^{2}-11.76X_{3}^{2}-12.18X_{4}^{2}$$

The ANOVA results for the model (Table 6) indicated that this model was highly significant (*P* < 0.0001). Furthermore, the lack of fit term showed a *P*-value of 0.9991, which was not significant, suggesting that the model captured all patterns in the data effectively and no other relationships affected the responses. The correlation coefficient (R^2^) is 0.9956, and the adjusted R^2^ is 0.9940, both of which demonstrated a good fit of the model to the data. These results confirmed that the model provided an excellent representation of the experimental data.

**TABLE 6:** ANOVA result for the model.

	Degree of freedom	Sum of squares	Mean of squares	F Value	*P* Value
FO (X1, X2, X3, X4)	4	1324.83	331.21	909.2719	<0.001
PQ (X1, X2, X3, X4)	4	413.94	103.48	284.0978	<0.001
Residuals	21	151.35	7.21		
Lack of fit	16	2.31	0.14	0.1353	0.9991
Pure error	5	5.34	1.07		

Note: The terms FO, and PQ, represent first-order and pure quadratic, respectively.

#### 3.1.3 The 3D response surface plot

**FIGURE 1:**
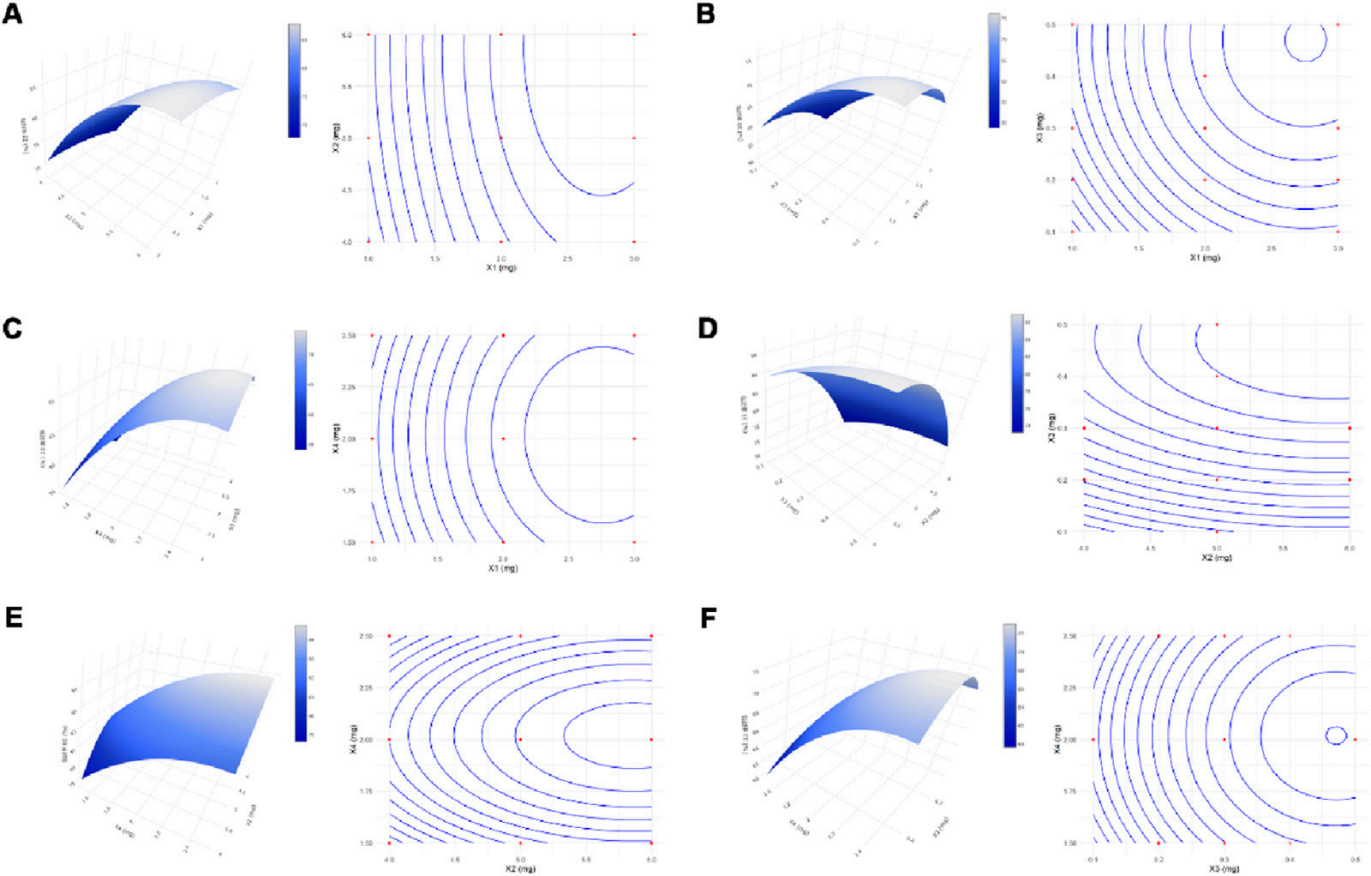
Response Surface and Contour Plots. Surface and contour plots demonstrated the effects of the interactions between variables X_1_ and X_2_ **(A)**, X_1_ and X_3_ **(B)**, X_1_ and X_4_ **(C)**, X_2_ and X_3_ **(D)**, X_2_ and X_4_ **(E)**, and X_3_ and X_4_ **(F)** on the response.

The response surface plots, including surface and contour plots, were further used to investigate the relationship between the independent variables and the response. In Figure 1A, the closely spaced contour lines along the X1 axis indicated that X1 had a more significant impact on EE%, while the sparser lines along X2 suggested a lesser effect. Figure 1B showed that sloped contours reflected a monotonic trend in EE%, with concentric arcs indicating possible local optimal EE% in mid-to-high factor levels. Figure 1C highlighted that high levels of X1 and X4 resulted in higher EE%, while Figure 1D demonstrated that EE% peaks when X2 was high and X3 was at a medium level. Figure 1E revealed an elliptical contour shape, suggesting a distinct peak, with a band-like inclination indicating monotonic changes in EE%. Lastly, Figure 1F showed concentric contours for X3 and X4, indicating a maximum or minimum EE% within specific ranges, with the contour lines’ slope reflecting a monotonic interaction between the factors.

#### 3.1.4 Formulation optimization result

After optimizing the micelle preparation conditions through both single-factor and response surface experiments, the optimal preparation conditions were determined as follows: X1 (DSPE-PEG-peptide) at 2.63 mg, X2 (PEG-PC7A) at 5.28 mg, X3 (MPLA) at 1.24 mg, and X4 (DOX) at 2.03 mg. To simplify the preparation process, these optimal conditions were adjusted to: X1 (DSPE-PEG-peptide) at 2.6 mg, X2 (PEG-PC7A) at 5.3 mg, X3 (MPLA) at 1.2 mg, and X4 (DOX) at 2.0 mg. Additionally, based on the results of the single-factor experiments, the other conditions were fixed as follows: organic solvent (chloroform/methanol 3:1, v/v) at 10 mL, and the hydration volume at 10 mL PBS (pH 7.4).

### 3.2 Characterizations of co-loaded nanoparticles

#### 3.2.1 Size, size distribution and zeta potential

**FIGURE 2:**
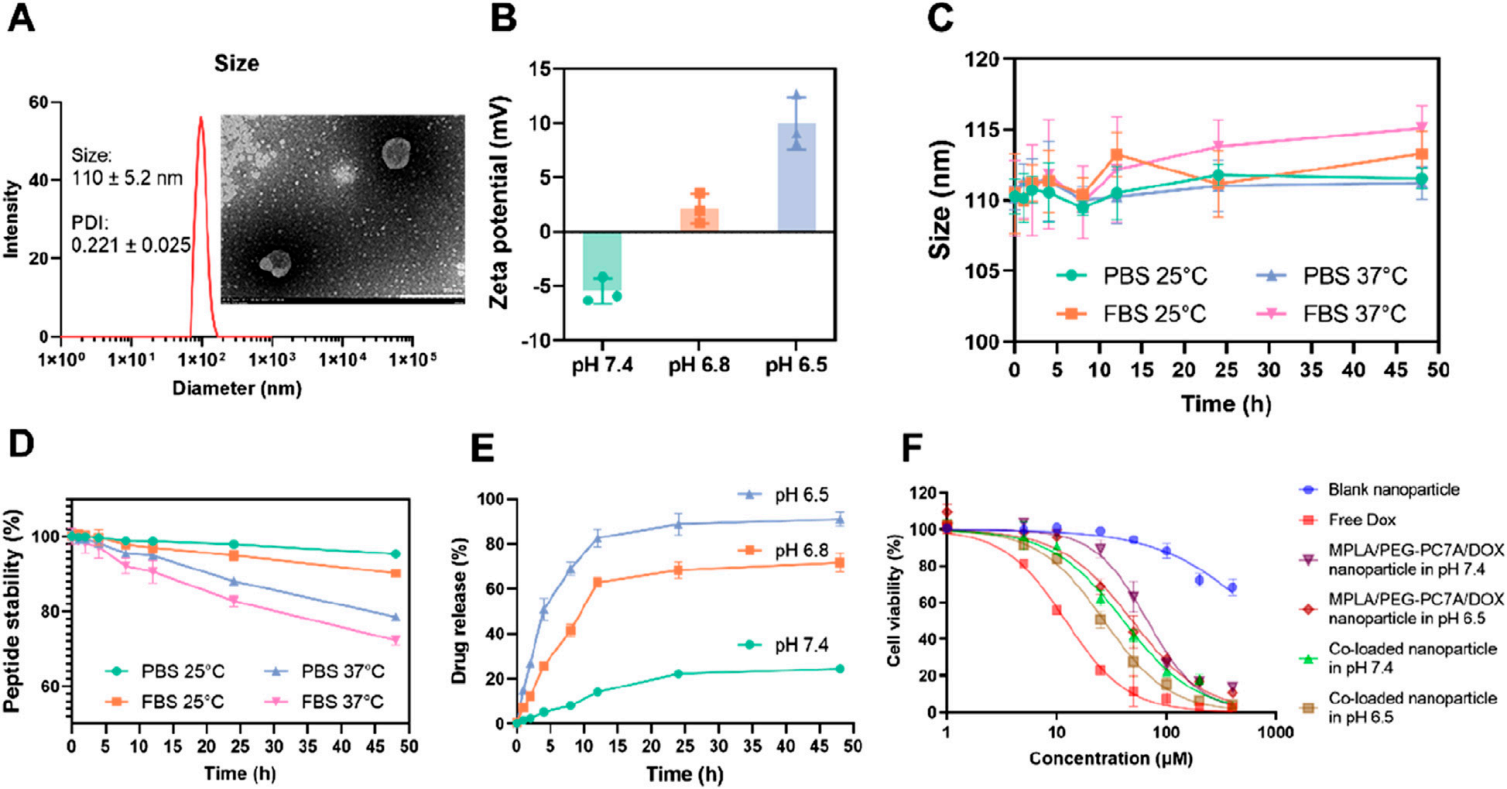
Characterization and stability of co-loaded nanoparticles. **(A)** Particle size distribution of co-loaded nanoparticles, with an inset showing the TEM image. The nanoparticles had a size of 110 ± 5.2 nm, and a polydispersity index (PDI) of 0.221 ± 0.025, consistent with the TEM result. **(B)** Zeta potential of the co-loaded nanoparticles under different pH conditions (7.4, 6.8, and 6.5). Data presented as mean ± SD (n = 3). **(C)** Particle size stability of co-loaded nanoparticles over time in PBS and FBS at 25°C and 37°C. Data presented as mean ± SD (n = 3). **(D)** The stability of PD-1/PD-L1 targeting peptide under different conditions. Data presented as mean ± SD (n = 3). **(E)** *In vitro* drug release profiles of DOX from the co-loaded nanoparticles at different pH values (7.4, 6.8, and 6.5). Data presented as mean ± SD (n = 3). **(F)** *In vitro* cytotoxicity measurement. The cell viability of LM8 cells treated with various groups for 48 h. Data are shown as mean ± SD (n = 3).

The optimal co-loaded nanoparticles were prepared based on the results stated above. Under these optimal conditions, the encapsulation efficiency of DOX was 97.15% ± 1.22%. As shown in Figure 2A, dynamic light scattering (DLS) analysis revealed that the co-loaded nanoparticles had an average particle size of 110 ± 5.2 nm with a polydispersity index (PDI) of 0.221 ± 0.025. The TEM image (inserted in Figure 2A) confirmed the morphology of the nanoparticles, which was in accordance with the DLS measurement. In terms of surface charge (Figure 2B), co-loaded nanoparticles exhibited a slight negative zeta potential under physiological environment, likely due to the minor negative charge of the peptide. However, in a mild acidic environment, the pH-sensitive PEG-PC7A undergoes a charge transition from neutral to positive, leading to a positive zeta potential at pH 6.8.

#### 3.2.2 Stability

To evaluate the stability of the co-loaded nanoparticles, the size of the nanoparticles was studied under different conditions. As shown in Figure 2C, the nanoparticles exhibited minimal size fluctuation when suspended in PBS, maintaining a stable diameter of approximately 110 nm over time. In contrast, when exposed to fetal bovine serum (FBS), a slight increase in particle size was observed, particularly at 37°C, where the size reached about 115 nm. This suggested moderate serum-induced aggregation, though the nanoparticles remained within an acceptable size range, indicating good colloidal stability.

To assess the stability of the conjugated peptide, the degradation profile of the peptide was investigated under different conditions (Figure 2D). In PBS at 37°C, the peptide remained over 90% intact after 48 h, indicating good stability in a physiological environment. However, in FBS-containing PBS, peptide degradation was more significant due to the proteolytic activity of serum proteins. Specifically, at 25°C, the peptide content decreased to 78.57% ± 0.81%, and further degradation was observed at 37°C, where the content reduced to 72.32% ± 1.26% after the same incubation period.

#### 3.2.3 *In vitro* drug release study

The release of doxorubicin from the nanoparticles was assessed in PBS solutions at pH 7.4, pH 6.8, and pH 6.5. As shown in Figure 2E, the drug release was slow under physiological conditions (pH 7.4), with a cumulative release of 24.38% ± 1.49% over 48 h. At pH 6.8, the hydrophobic-to-hydrophilic transition of PEG-PC7A induced a more rapid drug release, reaching 70.72% ± 4.22% at 48 h. At pH 6.5, further nanoparticle disassembly resulted in a significantly higher cumulative release of 91.08% ± 3.21%.

These results demonstrated that the co-loaded nanoparticles exhibited a pH-responsive drug release, facilitating the targeted co-delivery of multiple components within the tumor microenvironment, thereby supporting their potential for synergistic therapeutic application. Indeed, while the initial optimization efforts focused primarily on achieving best physicochemical properties, we recognize that these properties alone do not inherently guarantee optimal therapeutic outcomes. Consequently, the ultimate criterion for selecting our final formulation was the demonstration of antitumor activity and potent immune activation in both *in vitro* and *in vivo* experiments.

### 3.3 *In vitro* cellular studies

#### 3.3.1 *In vitro* cytotoxicity evaluation

To assess the cytotoxic effects of the co-loaded nanoparticles, LM8 murine osteosarcoma cells were incubated with different formulations, and cell viability was measured (Figure 2F). As a result, blank nanoparticles exhibited minimal cytotoxicity, indicating good biocompatibility of the carrier system. As a positive control, free DOX demonstrated potent cytotoxicity, with an IC_50_ of 13.27 ± 1.24 μM, confirming its strong anti-tumor effects. In contrast, the co-loaded nanoparticles displayed a sustained-release effect, as reflected by a higher IC_50_ of 40.63 ± 7.18 μM under physiological pH (pH 7.4). However, under the mildly acidic tumor microenvironment (pH 6.5), the IC_50_ decreased to 26.99 ± 2.64 μM, suggesting that the pH-responsive PEG-PC7A polymer facilitated nanoparticle disassembly, accelerating DOX release and enhancing cytotoxicity. To further demonstrate the influence of the PD-1/PD-L1-targeting peptide, additional nanoparticles containing only MPLA, PEG-PC7A and DOX (without peptide) were prepared and evaluated. Under physiological condition, these peptide-free nanoparticles displayed an IC_50_ value of 67.12 ± 2.32 μM, slightly higher than that of the fully co-loaded nanoparticles. At the mildly acidic environment (pH 6.5), the peptide-free nanoparticles exhibited an IC_50_ value of 48.76 ± 5.61 μM, which closely approximated the IC_50_ value of the fully co-loaded nanoparticles at physiological pH. These comparative results demonstrated that the incorporated peptide could enhance the recognition and interaction with PD-L1-positive tumor cells, thereby improving cytotoxicity and therapeutic efficacy. These results highlight that co-loaded nanoparticles effectively control drug release, exhibiting a pH-responsive cytotoxicity profile that ensures stability under physiological conditions while promoting rapid drug release in the tumor microenvironment, ultimately enhancing anti-tumor efficacy.

#### 3.3.2 Induction of immunogenic cell death by co-loaded nanoparticles

DOX is a well-established chemotherapeutic agent known for its ability to induce ICD, thereby triggering an anti-tumor immune response. During ICD induction, the externalization of calreticulin (CRT) and the release of high-mobility group box 1 protein (HMGB1) and adenosine triphosphate (ATP) serve as “eat me” and “danger” signals (Fucikova et al., 2021; Xi et al., 2024; Garg and Agostinis, 2017), respectively, to facilitate DC activation and immune response amplification. To evaluate whether the co-loaded nanoparticles can induce ICD in LM8 osteosarcoma cells, we assessed CRT exposure, ATP release, and HMGB1 secretion.

**FIGURE 3:**
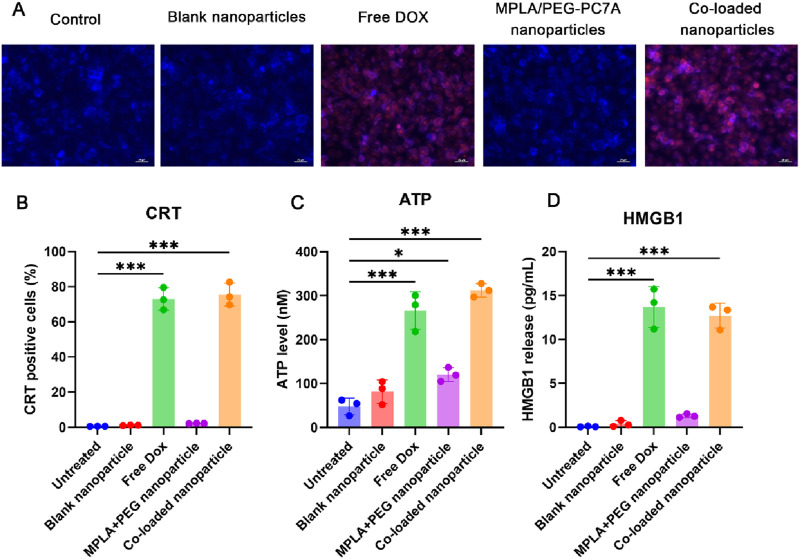
Induction of immunogenic cell death (ICD) in LM8 osteosarcoma cells by different formulations. **(A)** Immunofluorescence microscopy of CRT expression on the cell surface of LM8 cells after different treatments. The scale bar represents 50 μm. **(B)** CRT exposure analyzed by flow cytometry shows significant surface translocation in cells treated with free DOX and co-loaded nanoparticles. **(C)** ATP release quantified in the culture supernatant indicates strong ICD induction by free DOX and co-loaded nanoparticles, with minimal effects from other treatments. **(D)** HMGB1 release measured via ELISA confirms enhanced late-stage ICD signaling in free DOX- and co-loaded nanoparticle-treated cells. Data are presented as mean ± SEM, with statistical significance indicated as *p < 0.05, ***p < 0.001.

The exposure of CRT on the surface of LM8 was first studied using immunostaining analysis (Figure 3A). The results showed that the expression of CRT on the surface of LM8 cells treated with free DOX or co-loaded nanoparticles was significantly increased. To quantify the exposure of CRT, the flow cytometry was used. As shown in Figure 3B, the results were consistent with the immunostaining results. The untreated control, blank nanoparticles, and MPLA + PEG-PC7A nanoparticles alone did not induce significant CRT exposure in LM8 cells. However, treatment with free DOX led to CRT exposure in 73.04% ± 6.35% of cells, confirming its ICD-inducing capability. Notably, the co-loaded nanoparticles exhibited an even higher CRT exposure level of 75.54% ± 5.51%, indicating that the nanoparticles effectively preserved DOX’s ICD-inducing properties.

To further validate ICD induction, we quantified ATP release in the culture supernatant using an ATP detection assay (Figure 3C). The results demonstrated that the control, blank nanoparticles, and MPLA + PEG-PC7A nanoparticles alone had negligible effects on extracellular ATP levels. Although MPLA + PEG-PC7A nanoparticles induced a slight increase in ATP release, only free DOX and co-loaded nanoparticles significantly enhanced ATP secretion, with levels reaching 266.05 ± 42.73 nM and 312.2 ± 15.38 nM, respectively. These findings suggest that ATP release is primarily mediated by DOX-induced ICD, which is effectively maintained in the nanoparticle formulation.

The release of HMGB1 is also a crucial late-stage ICD marker (Fucikova et al., 2020), and it was assessed using an ELISA assay (Figure 3D). Similar to ATP release, only free DOX and co-loaded nanoparticles significantly increased HMGB1 secretion, with levels reaching 13.71 ± 2.31 ng/mL and 12.63 ± 1.42 ng/mL, respectively. This further confirms that the co-loaded nanoparticles successfully preserved the ICD-inducing capacity of DOX.

These results demonstrated that the co-loaded nanoparticles effectively retained and enhanced DOX-induced ICD properties, as evidenced by CRT exposure, ATP release, and HMGB1 secretion. These damage-associated molecular patterns (DAMPs) are critical in activating antigen-presenting cells and promoting adaptive immune responses. Notably, MPLA and PEG-PC7A alone did not induce significant ICD, suggesting that their primary function lies in immune activation rather than direct cytotoxicity. Additionally, since these treatments were given at an equivalent DOX concentration of 10 μM to allow direct comparison, this dose was near the efficacy threshold for free DOX in LM8 cells, which might underestimate the relative potency of the co-loaded nanoparticles. Due to the potential physicochemical properties, co-loaded nanoparticles might result in better ICD induction at low DOX concentration compared to free DOX treatment, which we will further confirm in future studies. Collectively, the ability of the co-loaded nanoparticles to simultaneously induce ICD and potentiate immune activation highlighted their potential as a multifunctional immunotherapeutic platform for osteosarcoma treatment.

#### 3.3.3 Activation of bone marrow-derived dendritic cells (BMDCs)

**FIGURE 4:**
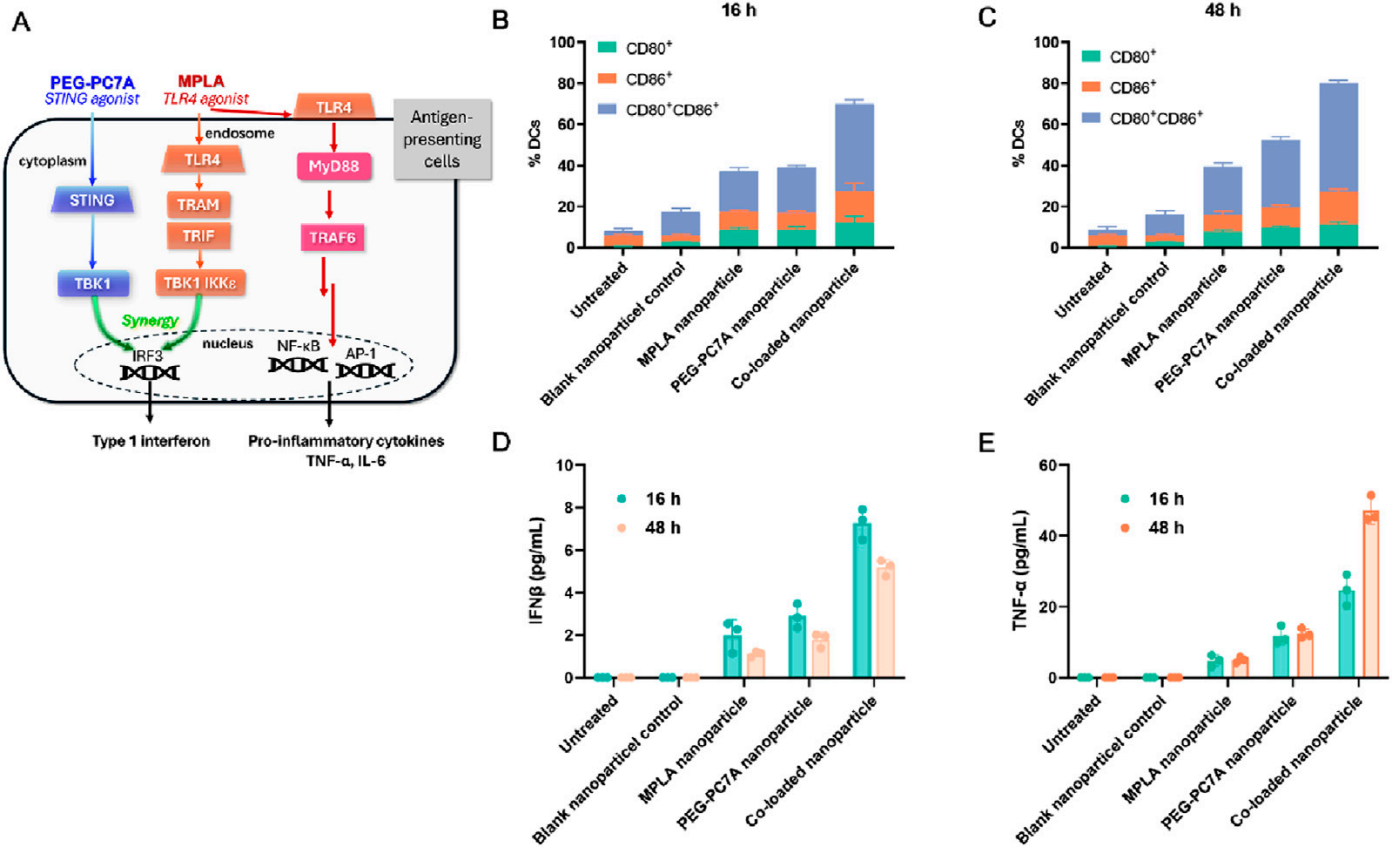
Effects of different groups on BMDC activation. **(A)** Schematic illustration of the mechanism of action, especially the synergistic activation of the STING and TLR4 pathways by PEG-PC7A and MPLA, respectively, leading to enhanced antigen presentation and cytokine secretion. **(B, C)** Expression levels of maturation markers CD80 and CD86 on BMDCs after 16 h **(B)** and 48 h **(C)** incubation with different groups. **(D, E)** Secretion levels of IFN-β **(D)** and TNF-α **(E)** in BMDCs at 16 h and 48 h, indicating STING and TLR4 pathway activation. Data are shown as mean ± SD (n = 3).

To investigate the immunostimulatory potential of MPLA and PEG-PC7A, BMDCs were stimulated with different formulations, including MPLA-only nanoparticles, PEG-PC7A-only nanoparticle, and co-loaded nanoparticles, and their maturation levels were analyzed at 16 h and 48 h post-incubation. As illustrated in Figure 4A, PEG-PC7A and MPLA synergistically enhance the production of type I interferons, including IFN-β. Upon the internalization of co-loaded nanoparticles, PEG-PC7A acts as a STING agonist, triggering the STING-TBK1-IRF3 signaling pathway (Basit et al., 2020; Corrales et al., 2016; Flood et al., 2019), which facilitates the production of IFN-β. Simultaneously, MPLA could activate the TLR4 TRIF-dependent pathway (Watts et al., 2017), further enhancing type I interferon secretion. The cooperative action of these pathways significantly amplifies immune responses, promoting BMDC activation and maturation. Additionally, MPLA could activate the MyD88-dependent TLR4 pathway on the cell surface (Hernandez et al., 2016; Ve et al., 2017), resulting in the activation of NF-κB and AP-1, which drive the secretion of pro-inflammatory cytokines such as TNF-α and IL-6. The interplay between these two pathways leads to a synergistic immune-stimulatory effect, promoting DCs maturation and cytokine release, thereby facilitating an improved anti-tumor immune response.

As shown in Figures 4B,C, compared to the control group, blank nanoparticles induced a mild activation of BMDCs, but the expression levels of CD80^+^CD86^+^ remained relatively low. Following incubation with MPLA or PEG-PC7A nanoparticles for 16 h, CD80^+^CD86^+^ expression increased to approximately 20%, suggesting moderate activation. In contrast, BMDCs treated with the co-loaded nanoparticles exhibited a significantly higher maturation level, with CD80^+^CD86^+^ expression reaching 41.52% ± 2.10%. After 48 h of incubation, all experimental groups showed an overall increase in DC maturation markers, with the co-loaded nanoparticle group demonstrating the most pronounced effect (CD80^+^CD86^+^ expression reaching 52.92% ± 1.44%), further confirming the sustained immune-activating potential of the formulation.

To assess the functional immune response, cytokine secretion was also evaluated. As shown in Figure 4D, IFN-β levels were moderately elevated in all treatment groups compared to the control. Notably, BMDCs treated with co-loaded nanoparticles secreted the highest level of IFN-β (7.28 ± 0.72 pg/mL) after 16 h incubation. Although IFN-β levels slightly declined to 5.19 ± 0.37 pg/mL after 48 h, they remained significantly higher than those observed in other treatment groups, suggesting sustained immune activation. Given that MPLA activates the TLR4/MyD88-dependent pathway, we also examined the secretion of TNF-α, a key pro-inflammatory cytokine (Figure 4E). The co-loaded nanoparticle group exhibited the highest levels of TNF-α, which increased over time, reaching 47.19 ± 3.75 pg/mL at 48 h incubation, showing a statistically significant difference compared to other groups.

Collectively, these results indicated that the co-loaded MPLA and PEG-PC7A nanoparticles could effectively enhance BMDC maturation and cytokine secretion through the synergistic activation of the TLR4 and STING pathways, demonstrating their strong potential as immune adjuvants. By integrating DOX-induced ICD with MPLA- and PEG-PC7A-mediated immune modulation, this strategy offers a promising approach to overcoming tumor immune evasion and enhancing anti-tumor immunity.

### 3.4 *In vivo* antitumor efficacy of co-loaded nanoparticles

#### 3.4.1 Tumor growth inhibition

**FIGURE 5:**
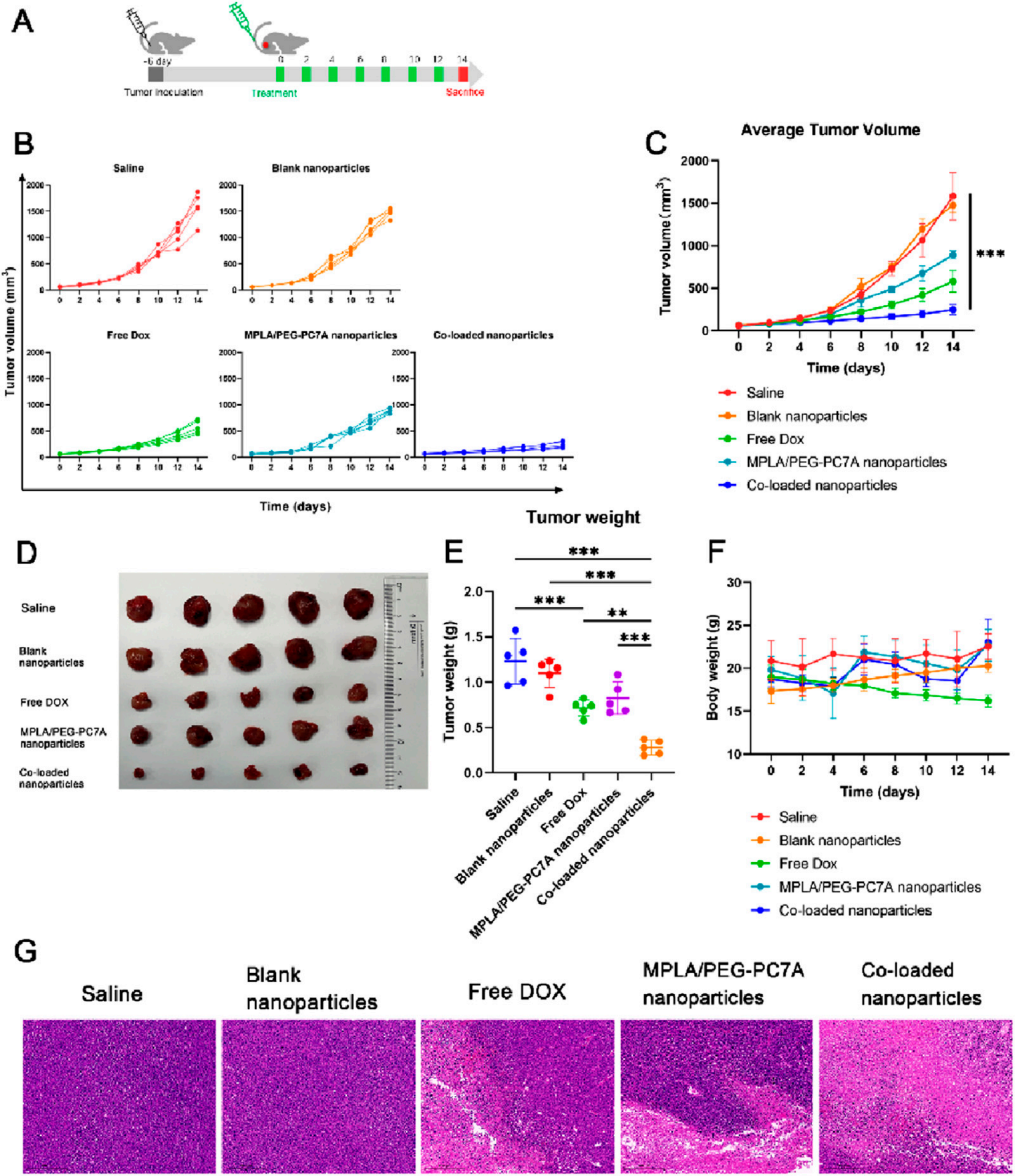
The therapeutic effect of co-loaded nanoparticles in LM-8-bearing tumors. **(A)** Schematic diagram of the experimental scheme. **(B)** Individual tumor growth curves f mice in different groups. **(C)** Average tumor sizes and standard error of the mean per group from experiment shown in panel **(B)**. Data represent means ± SD (n = 5). ***p < 0.001. **(D)** The digital images of tumors were collected from different groups of mice at the end of treatments. **(E)** Tumor weight in different groups at the end of treatment. Data represent means ± SD (n = 5). **p < 0.01; ***p < 0.001. **(F)** Body weights of the mice after various treatments. **(G)** Images of the tumor tissues stained with H&E collected from different experimental groups. Scale bar = 5 mm.

To evaluate the therapeutic efficacy of the multi-component co-loaded nanoparticles, an orthotopic LM-8 osteosarcoma model was established, and tumor growth was monitored over a 14-day treatment period (Figure 5A). Tumor volume progression for each treatment group is depicted in Figure 5B. Mice treated with saline or blank nanoparticles exhibited continuous tumor growth with no significant tumor suppression. In contrast, free DOX treatment resulted in a moderate reduction in tumor growth, while MPLA/PEG-PC7A nanoparticles demonstrated a less effective inhibitory effect compared to free DOX. Notably, the co-loaded nanoparticle group exhibited the most substantial tumor suppression, with minimal tumor progression observed throughout the study period. A comparative analysis of tumor volume (Figure 5C) revealed that treatment with co-loaded nanoparticles resulted in a significantly lower tumor volume compared to all other groups, highlighting the enhanced therapeutic efficacy of the combinational approach.

To further validate tumor inhibition, tumors were excised and weighed at the end of the study. Representative tumor images from each group are shown in Figure 5D. Consistent with tumor volume measurements, tumors from mice treated with saline or blank nanoparticles were the largest, whereas those treated with free DOX or MPLA/PEG-PC7A nanoparticles displayed moderate reductions in tumor mass. The co-loaded nanoparticle group demonstrated the most pronounced reduction in tumor size, providing clear evidence of superior antitumor efficacy. Quantitative tumor weight analysis (Figure 5E) showed a statistically significant reduction in tumor mass in the co-loaded nanoparticle group compared to all other treatment groups. These results further validate the potent antitumor activity of the co-loaded nanoparticles in suppressing osteosarcoma progression. Additionally, while we did not include a direct anti-PD-1 control group, the result indicated that the co-loaded nanoparticles provided significant benefits beyond those achieved by checkpoint blockade alone. This is particularly evident in our *in vitro* and *in vivo* experiments, where the co-loaded nanoparticles not only enhanced the cytotoxic effects of DOX but also induced ICD and stimulated immune responses through TLR4 and STING pathway activation. These mechanisms likely worked synergistically to amplify anti-tumor immunity, making our co-loaded nanoparticles more effective than conventional therapies that rely solely on checkpoint inhibitors. However, given the multi-modal nature of our therapeutic approach, it is important to assess the specific contributions of each mechanism. Therefore, future experiments will include a checkpoint inhibitor-only group to directly evaluate the relative contribution of checkpoint blockade to the observed therapeutic effects. This will provide a clear understanding of how much the combined immune-stimulating effects of the co-loaded nanoparticles and PD-1/PD-L1 blockade are responsible for the observed tumor suppression. This will also investigate the therapeutic benefit extends beyond what is achievable through checkpoint inhibition alone, highlighting the potential of multi-pronged immune modulation in overcoming tumor resistance.

The changes in body weight were monitored throughout the treatment to assess the systemic toxicity of nanoparticles. As shown in Figure 5F, treatment with free DOX led to significant weight loss in mice, indicating the severe toxicity of free DOX therapy. In contrast, mice received co-loaded nanoparticles maintained stable body weight and exhibited no apparent signs of systemic distress, indicating that our nanoparticles could reduce the systemic toxicity. While we did not perform an extensive cardiotoxicity analysis such as echocardiography or troponin levels, our co-loaded nanoparticles incorporating PEG-PC7A might be likely to provide protective benefits similar to those observed for pegylated liposomal DOX formulations, which have been known to reduce cardiac toxicity and alter DOX pharmacokinetics and distribution (Working et al., 1999; Rahman et al., 2007; Tahover et al., 2015). Moreover, the MPLA component within our co-loaded nanoparticles has been utilized at a dose within the safe range for vaccine adjuvants, and no acute inflammatory toxicities were observed. Future studies will systematically investigate these parameters, including cardiac histology, renal function, hematological profiles, and biochemical markers, to conclusively establish the comprehensive safety profile of our nanoparticle-based therapy.

To explore the pathological and microenvironmental changes in excised tumors, hematoxylin and eosin (H&E) staining was further performed. As shown in Figure 5G, a significantly reduced percentage of nuclei stained with hematoxylin in the co-loaded nanoparticle group was observed, implying the serious tumor destruction in mice.

These findings highlight the potential of co-loaded nanoparticles as a highly effective therapeutic strategy for osteosarcoma by integrating ICD-mediated chemotherapy, immune checkpoint blockade, and innate immune stimulation. Importantly, the optimal co-loaded nanoparticle formulation, which exhibited the best physicochemical properties, also demonstrated superior antitumor efficacy in both cellular and animal models. This result showed the successful translation of optimized physical parameters into significantly enhanced therapeutic performance.

#### 3.4.2 Immune cell profiling in the tumor microenvironment

To further investigate the immune-modulating effects of the co-loaded nanoparticles, immune cell populations in the tumor microenvironment were analyzed post-treatment using flow cytometry. Key immune parameters, including DC maturation, CTL infiltration, effector T cell activation, memory T cell formation, and Treg suppression, were systematically assessed.

**FIGURE 6:**
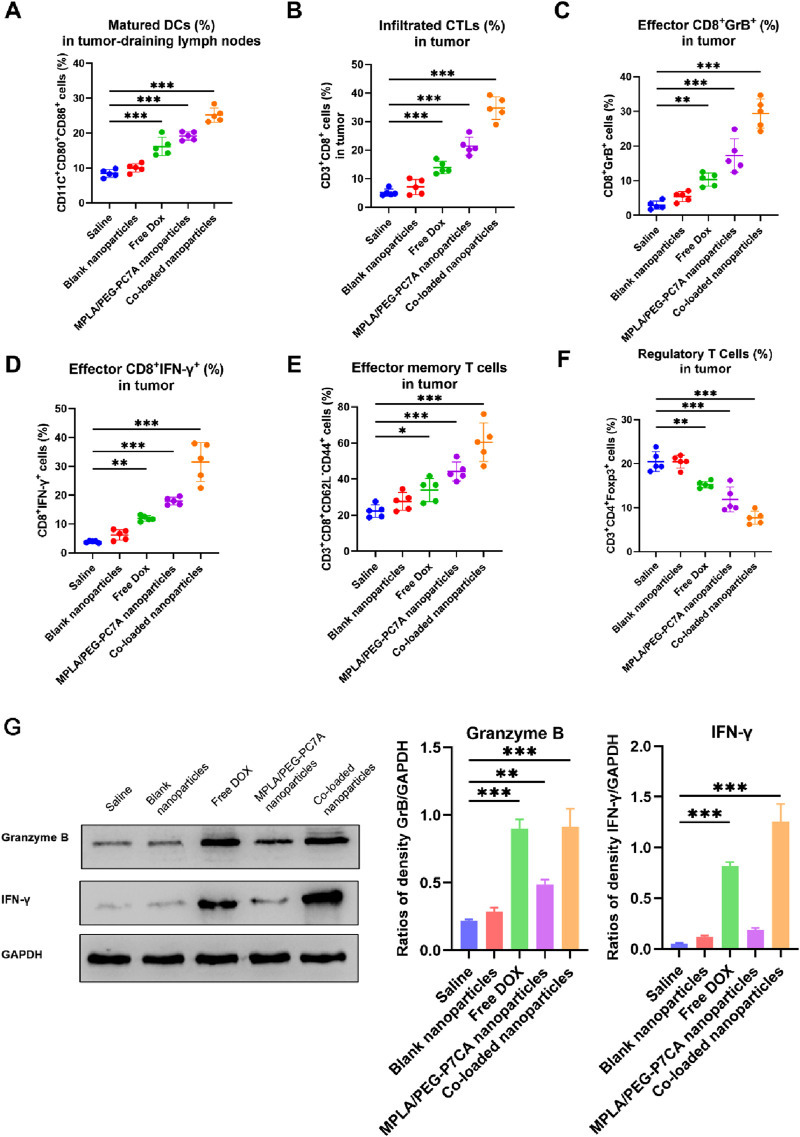
Immune cell profiling in the tumor microenvironment after treatment with different groups. **(A)** Percentage of mature DCs (CD11c^+^CD80^+^CD86^+^) in the tumor-draining lymph nodes, indicating enhanced antigen presentation. **(B)** Infiltrated cytotoxic T lymphocytes (CTLs) (CD3^+^CD8^+^) in the tumor tissue, reflecting improved T cell recruitment to the tumor site. **(C)** Effector CD8^+^ T cells expressing Granzyme B (GrB^+^) in the tumor tissue, indicating cytolytic potential. **(D)** Effector CD8^+^ T cells expressing IFN-γ^+^ in the tumor tissue, demonstrating enhanced immune activation. **(E)** Effector memory T cells (CD3^+^CD8^+^CD62L^−^CD44^+^) in the tumor tissue, highlighting the potential for long-term anti-tumor immunity. **(F)** Regulatory T cells (Tregs) (CD3^+^CD4^+^ Foxp3^+^) in the tumor tissue, showing a reduction in immunosuppressive cells. In panels A–E, data are presented as mean ± SEM, with statistical significance indicated as *p < 0.05, **p < 0.01, ***p < 0.001. **(G)** Western blotting. The representative images and quantitative data of Granzyme B and IFN-γ in tumor tissues after treatments (n = 4). One-way ANOVA with Turkey’s multiple comparison test, with statistical significance indicated as *p < 0.05, **p < 0.01, ***p < 0.001.

Mature DCs (CD11c^+^CD80^+^CD86^+^) play a critical role in antigen presentation and the activation of adaptive immune responses. As shown in Figure 6A, treatment with co-loaded nanoparticles significantly enhanced DC maturation in the tumor-draining lymph nodes compared to free DOX and MPLA/PEG-PC7A nanoparticles. This enhancement was likely due to the combined effects of MPLA-mediated TLR4 activation, PEG-PC7A-mediated STING activation, and DOX-induced ICD. MPLA stimulated DCs maturation through TLR4 signaling, while PEG-PC7A activated the STING pathway, leading to type I interferon (IFN-β) production, which further promoted DC maturation and enhanced antigen presentation.

Effective tumor immunity relies on the infiltration of CD8^+^ cytotoxic T lymphocytes (CTLs) into the tumor microenvironment. In terms of CTLs infiltration (Figure 6B), free DOX and MPLA/PEG-PC7A nanoparticles promoted moderate CTLs infiltration, while the co-loaded nanoparticles demonstrated the highest percentage of tumor-infiltrating CTLs. Further analysis revealed that a significant increase in effector CD8^+^ T cells expressing granzyme B (GrB^+^) and interferon-γ (IFN-γ^+^) as well as effector memory T cells (CD3^+^CD8^+^CD62L^−^CD44^+^) in the co-loaded nanoparticle group (Figures 6C–E). This enhancement is likely attributed to the checkpoint blockade provided by the PD-1/PD-L1 targeting peptide, which prevented T cell exhaustion and sustained CTL activity. The combination of DOX-induced ICD and immune checkpoint blockade resulted in a significant increase in effector memory T cells in tumor-bearing mice. The presence of these memory T cells is a key indicator of long-term adaptive immunity and demonstrated that the co-loaded nanoparticles not only activated an acute immune response to combat the primary tumor but also effectively educated the immune system to recognize tumor antigens. This priming allows the immune system to remain poised for rapid reactivation in response to any residual or recurring tumor cells, thereby potentially reducing the risk of tumor relapse. Moreover, IFN-β and other cytokines induced by co-loaded nanoparticles could facilitate the cross-priming of T cells and enhance DC maturation, which are known to contribute to the formation of durable anti-tumor immunity. These effects align well with our data on enhanced DC maturation (Figure 6A), further demonstrating the potential of our therapy to achieve sustained anti-tumor immunity. In the future, we will conduct a tumor rechallenge experiment to investigate long-term immunity after the treatment of co-loaded nanoparticles, for example, curing mice of their tumors with our therapy and then re-injecting them with OS cells at a later time to see if the immune system prevents tumor regrowth.

Tregs (CD3^+^CD4^+^Foxp3^+^) play a key role in maintaining immune tolerance and suppressing anti-tumor immunity. A reduction in Tregs is often associated with enhanced immune activation. As shown in Figure 6F, treatment with co-loaded nanoparticles resulted in the most significant depletion of Tregs. This reduction likely reflected the synergistic immune-stimulatory effects of MPLA, PEG-PC7A, and the PD-1/PD-L1-targeting peptide, which synergistically inhibited immune suppression and promoted an effective anti-tumor response. By reducing the population of Treg cells and other suppressive factors, the treatment might allow the activated effector T cells and memory T cells to persist longer and function more effectively, thereby prolonging the immune response even after therapy.

Western blot analysis of tumor tissues confirmed a significant increase in the levels of granzyme B and IFN-γ in the co-loaded nanoparticle group (Figure 6G). In comparison to the saline group, granzyme B levels increased by 3.18-fold, and IFN-γ levels increased by 19.4-fold. The results indicated the efficacy of PD-1/PD-L1 immune checkpoint blockade, consistent with the flow cytometry data.

These results highlight that the superior anti-tumor efficacy of the co-loaded nanoparticles was driven by the synergistic combination of DOX-induced ICD, MPLA-mediated TLR4 activation, PEG-PC7A-activated STING pathway, and PD-1/PD-L1 blockade. Taken together, these mechanisms promoted DC maturation, CTLs activation, Treg depletion, and long-term immune surveillance, effectively reprogramming the tumor microenvironment from immunosuppressive to immunostimulatory and enhancing therapeutic efficacy. Therefore, the co-loaded nanoparticles achieving optimal physicochemical properties also demonstrated superior anti-tumor efficacy *in vitro* and *in vivo*, suggesting a good translation from those properties and therapeutic performance.

## 4 Conclusion

In conclusion, we designed a multi-component co-loaded nanoparticles as a highly effective immunochemotherapeutic strategy for osteosarcoma. By combining ICD-mediated chemotherapy, TLR4-STING co-activation, and PD-1/PD-L1 blockade, this system not only enhanced immediate tumor cytotoxicity but also established a long-lasting anti-tumor immune memory. The multi-faceted immune modulation conferred by this approach highlights its promise for clinical translation in the treatment of osteosarcoma and other immunoresistant malignancies.

## Data Availability

The original contributions presented in the study are included in the article/Supplementary Material, further inquiries can be directed to the corresponding authors.
