# Supplementary material for: Synergistic chemo-immunotherapy for osteosarcoma via a pH-responsive multi-component nanoparticle system
Source: Front Pharmacol. 2025 Apr 8;16:1584245. doi: 10.3389/fphar.2025.1584245 (PMC12011790; doi:10.3389/fphar.2025.1584245)
Supplement: Supplementary file 1 [file DataSheet1.docx]

**Supplementary Material**

Synergistic chemo-immunotherapy for osteosarcoma via a pH-responsive multi-component nanoparticle system

Figure S1. ^1^H NMR spectrum of PEG-PC7A.

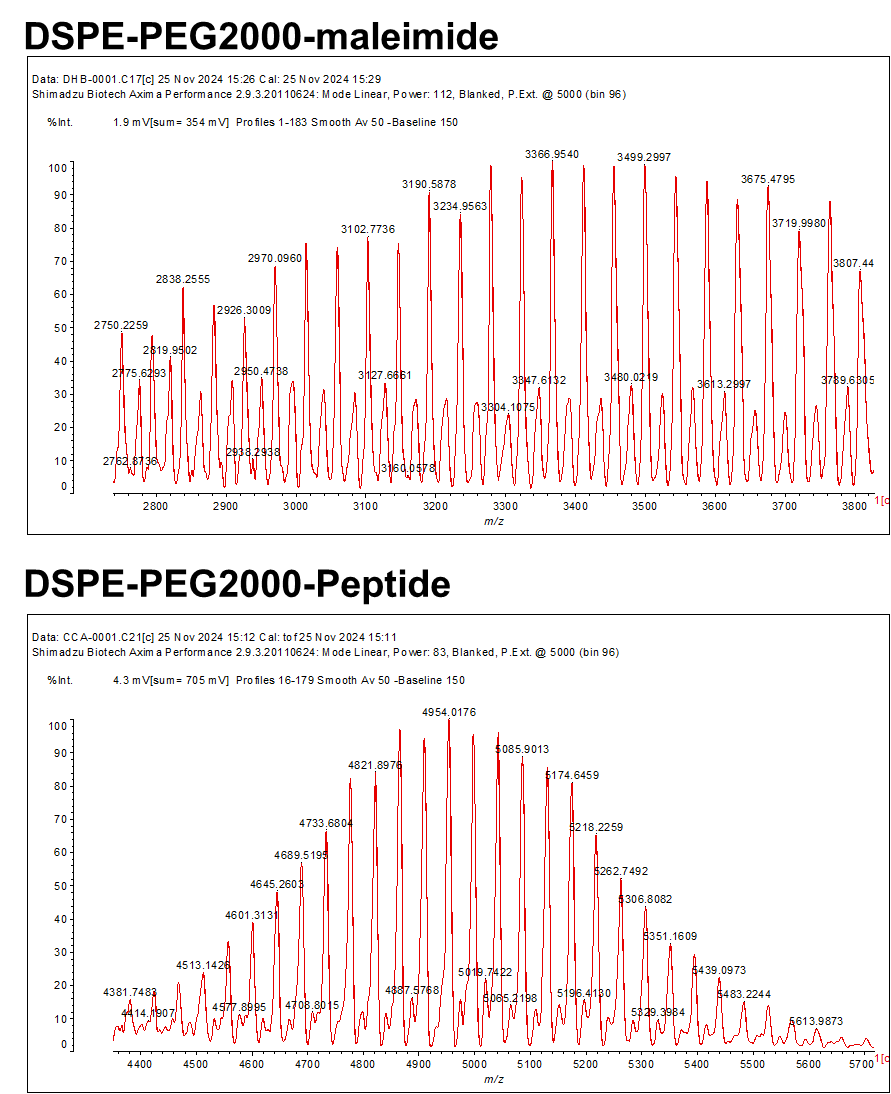


Figure S2. MALDI-TOF-MS spectrum of DSPE-PEG-Mal and DSPE-PEG-Peptide.

Table S1. The results of BBD

| Run order | Variables | | | | Y (EE%) | |
| --- | --- | --- | --- | --- | --- | --- |
|  | X1 | X2 | X3 | X4 | Experimental | Predicted |
| 1 | 1 | 4 | 0.2 | 1.5 | 62.58 | 62.48 |
| 2 | 1 | 4 | 0.2 | 2.5 | 63.23 | 63.16 |
| 3 | 1 | 6 | 0.2 | 1.5 | 64.99 | 64.68 |
| 4 | 1 | 6 | 0.2 | 2.5 | 65.67 | 65.36 |
| 5 | 3 | 4 | 0.2 | 1.5 | 78.13 | 77.73 |
| 6 | 3 | 4 | 0.2 | 2.5 | 78.48 | 78.4 |
| 7 | 3 | 6 | 0.2 | 1.5 | 79.99 | 79.93 |
| 8 | 3 | 6 | 0.2 | 2.5 | 80.81 | 80.6 |
| 9 | 1 | 5 | 0.1 | 2 | 65.97 | 66.34 |
| 10 | 3 | 5 | 0.5 | 2 | 90.47 | 90.43 |
| 11 | 1 | 5 | 0.5 | 2 | 75.50 | 75.18 |
| 12 | 3 | 5 | 0.1 | 2 | 81.60 | 81.59 |
| 13 | 2 | 4 | 0.3 | 1.5 | 78.57 | 78.46 |
| 14 | 2 | 4 | 0.3 | 2.5 | 79.15 | 79.14 |
| 15 | 2 | 6 | 0.3 | 1.5 | 80.61 | 80.66 |
| 16 | 2 | 6 | 0.3 | 2.5 | 81.25 | 81.34 |
| 17 | 2 | 5 | 0.2 | 1.5 | 78.74 | 79.17 |
| 18 | 2 | 5 | 0.4 | 1.5 | 83.10 | 83.59 |
| 19 | 2 | 5 | 0.2 | 2.5 | 79.48 | 79.85 |
| 20 | 2 | 5 | 0.4 | 2.5 | 84.04 | 84.27 |
| 21 | 1 | 4 | 0.3 | 2 | 67.73 | 68.19 |
| 22 | 1 | 6 | 0.3 | 2 | 70.11 | 70.39 |
| 23 | 3 | 4 | 0.3 | 2 | 83.12 | 83.44 |
| 24 | 3 | 6 | 0.3 | 2 | 85.17 | 85.64 |
| 25 | 2 | 5 | 0.3 | 2 | 83.79 | 84.88 |
| 26 | 2 | 5 | 0.3 | 2 | 84.19 | 84.88 |
| 27 | 2 | 5 | 0.3 | 2 | 84.85 | 84.88 |
| 28 | 2 | 5 | 0.3 | 2 | 86.40 | 84.88 |
| 29 | 2 | 5 | 0.3 | 2 | 85.65 | 84.88 |
| 30 | 2 | 5 | 0.3 | 2 | 85.96 | 84.88 |
